# Safety and Feasibility of Same-Day Discharge After Balloon Pulmonary Angioplasty for Chronic Thromboembolic Pulmonary Hypertension: A Single-Center Retrospective Study

**DOI:** 10.1016/j.jscai.2026.105505

**Published:** 2026-07-16

**Authors:** Kevin Jin, Kelley Chen, Benjamin L. Magod, Zhiying Meng, Heather Byrd, Abigail S. Baldridge, Daniel J. Romary, Benjamin H. Freed, Yasmin Raza, Maanasi Samant, Ruben Mylvaganam, Michael J. Cuttica, S. Chris Malaisrie, Stephen F. Chiu, Daniel R. Schimmel

**Affiliations:** a Division of Cardiac Surgery, Northwestern University Feinberg School of Medicine and Bluhm Cardiovascular Institute at Northwestern Medicine, Chicago, Illinois; b Division of Cardiology, Northwestern University Feinberg School of Medicine and Bluhm Cardiovascular Institute at Northwestern Medicine, Chicago, Illinois; c Department of Internal Medicine, Northwestern University Feinberg School of Medicine, Chicago, Illinois; d Division of Pulmonary & Critical Care, Northwestern University Feinberg School of Medicine and Canning Thoracic Institute at Northwestern Medicine, Chicago, Illinois

**Keywords:** balloon pulmonary angioplasty, chronic thromboembolic pulmonary hypertension, same-day discharge

## Abstract

**Background:**

Balloon pulmonary angioplasty (BPA) is increasingly used for patients with inoperable or residual chronic thromboembolic pulmonary hypertension. Overnight observation is the current standard practice. We evaluated the safety and feasibility of same-day discharge (SDD) after BPA at a contemporary US referral center.

**Methods:**

We retrospectively analyzed consecutive chronic thromboembolic pulmonary hypertension patients undergoing BPA between 2018 and 2024. SDD was defined as discharge on the calendar day of BPA. The primary outcome was 30-day readmission or mortality; secondary outcomes included BPA-related complications. Predictors of SDD were assessed using mixed-effects logistic regression.

**Results:**

Seventy-seven patients underwent 262 BPA procedures; 89 (34%) were followed by SDD. Patients selected for SDD had higher baseline 6-minute walk distance (356 vs 270 m; *P* = .010). SDD procedures were shorter (89 vs 101 minutes; *P* < .001) and associated with lower mean pulmonary artery pressure (28.5 vs 34.9 mm Hg; *P* < .001). Overall, 30-day complication rates were low (6/262, 2.3%), with no periprocedural deaths. Thirty-day readmission rates were similar between SDD and non-SDD procedures (7.9% vs 4.0%; *P* = .43). In multivariable analysis, higher 6-minute walk distance (odds ratio [OR], 1.66 per 100 m; *P* = .026), device closure (OR, 6.33; *P* = .028), and larger balloon size (OR, 1.44 per mm; *P* = .037) were associated with SDD, whereas initial BPA session (OR, 0.13; *P* < .001) and longer procedure duration (OR, 0.66 per 10 minutes; *P* < .001) were associated with admission.

**Conclusions:**

Among patients meeting prespecified institutional eligibility criteria, SDD after BPA was feasible and not associated with increased 30-day adverse events. Structured SDD pathways guided by reproducible selection criteria may optimize resource utilization as BPA volumes continue to grow.

## Introduction

Chronic thromboembolic pulmonary hypertension (CTEPH) arises when thromboembolic material from an acute pulmonary embolism fails to resolve and instead organizes into chronic, fibrotic obstruction of the pulmonary arteries.^1^^,^^2^ Left untreated, CTEPH can lead to right heart failure and ultimately death.^3^

Balloon pulmonary angioplasty (BPA) is the treatment of choice for patients with CTEPH who are not candidates for pulmonary thromboendarterectomy (PTE). The number of centers performing PTE surgery is growing in the United States (US); however, candidacy for surgery varies by center expertise, and a subset of patients are deemed inoperable because of distal disease distribution or prohibitive surgical risk.^4^ In addition, some patients opt not to undergo surgery or remain with residual pulmonary hypertension after PTE.^1^ In these cases, patients are best served with BPA if anatomically feasible.^1^^,^^5^ BPA procedural volumes have increased over the past decade in Japan, Europe, and the US, reflecting the growing role of BPA for the management of inoperable and residual CTEPH.^2^^,^^6^, ^7^, ^8^

Balloon pulmonary angioplasty has traditionally been followed by overnight inpatient observation because of concerns for procedure-related complications such as hemoptysis, reperfusion lung injury, and hypoxemia.^6^^,^^9^ However, contemporary BPA techniques emphasize staged treatment, conservative lesion dilation, and improved periprocedural monitoring, which may mitigate these risks. Despite these advances, postprocedural protocols following BPA have not evolved in parallel, and overnight hospitalization remains standard practice at many centers, a practice largely informed by international experience from Japan and Europe.^1^^,^^7^^,^^10^^,^^11^ As BPA procedural volumes continue to rise, routine overnight hospitalization may unnecessarily strain inpatient resources, underscoring the need for evidence-based discharge strategies.

Across interventional cardiology, same-day discharge (SDD) pathways have been adopted and shown to safely reduce unnecessary overnight observation without compromising clinical outcomes, which have consequently improved hospital bed availability and staffing efficiency for higher-acuity care.^12^ For instance, SDD has been successfully implemented after elective percutaneous coronary intervention^13^^,^^14^ and transcatheter aortic valve replacement.^15^^,^^16^ These successes have prompted reconsideration of routine overnight observation following other complex catheter-based procedures, including BPA.

This study aims to evaluate the safety and feasibility of SDD following BPA in a contemporary US cohort selected by a prespecified institutional eligibility algorithm and to identify clinical and procedural characteristics of patients who underwent successful SDD.

## Materials and methods

### Study design and setting

This was a retrospective cohort study conducted at Northwestern Memorial Hospital, a comprehensive CTEPH referral center. We included all patients with CTEPH who underwent BPA between January 8, 2018, and December 30, 2024, and who consented to be included in an institutional registry. This study was approved by the institutional review board (IRB #STU00009959).

The institution operates a multidisciplinary comprehensive CTEPH program with active PTE and BPA programs and a standing multidisciplinary team meeting, including interventional cardiology, cardiothoracic surgery, pulmonary hypertension physicians, and radiologists.

### Patient population

All consecutive adult patients (≥18 years) with CTEPH who underwent at least 1 BPA procedure and consented to study participation during the study period were included. The diagnosis of CTEPH and patient eligibility for BPA or PTE were determined through multidisciplinary evaluation.

Baseline characteristics, including demographic characteristics, comorbidities, imaging findings, echocardiographic parameters, and functional status, were obtained from the medical record at the time of each patient’s first BPA procedure. A total of 77 patients undergoing 262 BPA procedures were included. Patients who had at least 1 SDD following BPA were the case group, and patients who did not have SDD were the control group.

### Procedure details

All BPA procedures were performed using standard catheter-based techniques under fluoroscopic guidance. Vessels were selected based on pulmonary angiography and preprocedural imaging.

Procedural variables included lobar and segmental distribution of intervention, number of treated segments per procedure, largest balloon diameter used, contrast volume, radiation dose, procedure duration, and hemostasis strategy (manual compression, closure device, or mattress suture). Invasive periprocedural hemodynamics, including mean pulmonary artery pressure (mPAP), pulmonary vascular resistance (PVR), and cardiac output, were recorded when available.

### Discharge criteria

Discharge disposition was determined using a standardized institutional algorithm. SDD was defined as discharge on the calendar day of BPA.

Patients were eligible for SDD if they met all predefined criteria: (1) Historical criteria: age <75 years; left ventricular ejection fraction >40%; supplemental oxygen requirement ≤2 L/min; no mobility assistance device; no contrast intolerance; patient agreement with SDD; second or subsequent BPA session with no prior hemoptysis or major complication; and supervision at home for 24 hours. (2) Postprocedural criteria: procedure completed before 1:00 PM; no increase in oxygen requirement; no hemoptysis or acute complication; no access-site hematoma; ≥2-hour observation after sheath removal; and clearance by the interventional cardiologist and nurse clinician.

These criteria were used as a framework rather than absolute requirements. In select cases, patients who did not meet all historical criteria were discharged the same-day at the discretion of the procedural team if the session was limited in scope (eg, few segments treated), technically straightforward, and without intraprocedural complications. Conversely, patients meeting criteria could still be observed overnight based on clinical judgment.

All SDD patients received standardized discharge instructions, direct physician contact information, and next-day telephone follow-up. Patients requiring planned admission or requiring overnight monitoring were classified as non-SDD.

### Outcomes

The primary outcome measure was readmission or mortality within 30 days of BPA. Secondary outcome measures were BPA-related complications within 30 days. These included hypoxemic respiratory failure (including reperfusion lung injury), access-site complications, pneumonia, syncope, or hemorrhagic events.

### Statistical analysis

Baseline and procedural characteristics were summarized using descriptive statistics. Mean and SD were used for normally distributed continuous variables. Median with interquartile range (IQR) were used for nonnormally distributed variables. Frequencies with percentages were used for categorical variables. Patient-level summaries of the baseline characteristics reflected data of the first procedure if patients had multiple BPA and were compared between the SDD group and the control group using 2-sample *t* tests, Wilcoxon rank-sum tests, χ^2^ test, or Fisher exact tests as appropriate. Procedural-level comparisons between groups were performed using univariable mixed-effect logistic models to account for within-subject correlation, considering some patients had multiple BPA procedures. Comparison of 30-day complication rates between the SDD and non-SDD groups was performed using a generalized estimating equation at the procedure-level.

Multivariable mixed-effect logistic regression analysis was performed to evaluate the associations of baseline and procedural characteristics with SDD. The following variables were included in the model: age, PVR group, and other baseline and procedural variables with *P* <.05 in the univariable analysis. Continuous predictors were scaled to clinically meaningful units (eg, 6MWD per 100 meters, procedure duration per 10 minutes, balloon diameter per 1 mm). Odds ratio (OR) with 95% CI are reported. Analyses were performed using SAS version 9.4 (SAS Institute) and R (The R Project for Statistical Computing), with 2-sided *P* values <.05 considered statistically significant.

## Results

### Patient Characteristics

**Figure 1:**
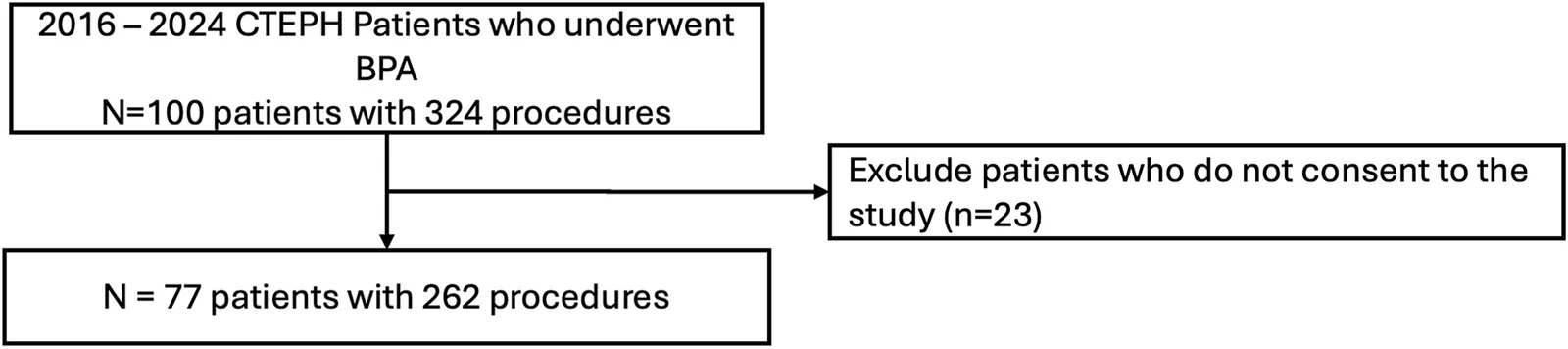
**Study cohort and procedural flow.** BPA, balloon pulmonary angioplasty; CTEPH, chronic thromboembolic pulmonary hypertension.

Between 2018 and 2024, 77 unique patients underwent a total of 262 BPA procedures (Figure 1). Nearly two-thirds (n = 47; 61%) had at least 1 SDD, and the remaining (n = 30; 39%) never had an SSD. The mean age of the cohort was 61.7 ± 14.7 years, and 48 patients (62.3%) were female. The overall cohort had a substantial cardiopulmonary disease burden: 38 patients (49.4%) had at least mild right ventricular dysfunction on baseline echocardiography, and 34 (44.2%) required supplemental oxygen prior to their first BPA. Twenty patients (26.0%) were not candidates for PTE because of distal disease or prohibitive surgical risk, while 40 (51.9%) had previously undergone PTE. The remaining 17 patients (22.1%) were operative candidates who opted not to undergo surgery (Table 1). Baseline data were complete for most variables. Left ventricular ejection fraction was unavailable for 2 patients, and preprocedure 6-minute walk distance (6MWD) was documented for 66 of 77 patients (85.7%).

**Table 1:** Baseline characteristics of patients undergoing BPA

Characteristic	All patients (N = 77)	Ever experienced same-day discharge		
		No (n = 30)	Yes (n = 47)	*P* value^a^
Age, y	61.7 ± 14.7	62.2 ± 15.1	61.4 ± 14.6	.81
Female	48 (62.3)	20 (66.7)	28 (59.6)	.53
Body mass index, kg/m^2^	33.7 ± 9.1	34.3 ± 10.3	33.3 ± 8.4	.65
Race/ethnicity				.51
Caucasian	51 (66.2)	20 (66.7)	31 (66.0)	
African American	20 (26.0)	8 (26.7)	12 (25.5)	
Hispanic	3 (3.9)	2 (6.7)	1 (2.1)	
Other	3 (3.9)	0 (0)	3 (6.4)	
Procedural characteristics				
Total no. of BPA sessions	3 (2-4)	3 (2-4)	3 (2-5)	.33
Reasons for BPA				.41
Not a surgical candidate	20 (26.0)	7 (23.3)	13 (27.7)	
Post-PTE	40 (51.9)	14 (46.7)	26 (55.3)	
Patient preference	17 (22.1)	9 (30.0)	8 (17.0)	
Cardiac history and risk factors				
Contrast allergy	3 (3.9)	2 (6.7)	1 (2.1)	.56
HFrEF (EF < 40%)	8 (10.4)	2 (6.7)	6 (12.8)	.47
Coronary artery disease	30 (39.0)	12 (40.0)	18 (38.3)	.88
Type II diabetes mellitus	13 (16.9)	6 (20.0)	7 (14.9)	.56
Hypertension	45 (58.4)	19 (63.3)	26 (55.3)	.49
History of smoking	25 (32.5)	13 (43.3)	12 (25.5)	.11
LVEF, %	62.1 ± 7.1	62.3 ± 7.9	62.0 ± 6.7	.85
RV dysfunction				.16
None	39 (50.6)	11 (36.7)	28 (59.6)	
Mild	17 (22.1)	7 (23.3)	10 (21.3)	
Moderate	8 (10.4)	4 (13.3)	4 (8.5)	
Severe	13 (16.9)	8 (26.7)	5 (10.6)	
Required oxygen	34 (44.2)	20 (66.7)	14 (29.8)	.002
eGFR, mL/min/1.73 m^2^				.89
≥60	61 (79.2)	24 (80.0)	37 (78.7)	
<60	16 (20.8)	6 (20.0)	10 (21.3)	
Preprocedure 6MWD, m	326 ± 132	270 ± 120	356 ± 130	.010
Medication				
Riociguat	33 (42.9)	14 (46.7)	19 (40.4)	.59
Anticoagulation				.70
DOAC	45 (58.4)	17 (56.7)	28 (59.6)	
Warfarin	29 (37.7)	11 (36.7)	18 (38.3)	
Other	3 (3.9)	2 (6.7)	1 (2.1)	

Values are either mean ± SD, n (%), or median (IQR).

6MWD, 6-minute walk distance; BPA, balloon pulmonary angioplasty; DOAC, direct oral anticoagulant; EF, ejection fraction; eGFR, estimated glomerular filtration rate; HFrEF, heart failure with reduced ejection fraction; LVEF, left ventricular ejection fraction; RV, right ventricular.

a *P* value was determined by χ^2^ test, Fisher exact test, 2 sample *t* test, or Wilcoxon test, as appropriate. Values reflect data of the first BPA procedure if patients had multiple BPA. LVEF was unavailable for 2 patients, and preprocedure 6-minute walk distance was documented for 66 of 77 patients.

Baseline demographic characteristics, including age, sex, body mass index, and ethnicity, were similar between the SDD and non-SDD groups. Both groups also had comparable total numbers of BPA sessions and similar indications for BPA.

Comorbid conditions and cardiac characteristics, including coronary artery disease, heart failure with reduced ejection fraction, diabetes, hypertension, left ventricular ejection fraction, baseline right ventricular dysfunction severity, renal function, and contrast allergy, did not differ significantly between groups. Pulmonary hypertension medical therapy and anticoagulation strategy were also similar.

Patients in the non-SDD group had a higher incidence of supplemental oxygen prior to their first BPA (66.7% vs 29.8%, *P* = .002) and had significantly lower baseline 6MWD (270 ± 120 m vs 356 ± 130 m, *P* < .001) (Table 1).

### Procedural characteristics

Among the 262 BPA procedures included, 89 (34.0%) were followed by SDD, and 173 (66.0%) required overnight or longer hospitalization (Table 2, Central Illustration). SDD was significantly less common during initial BPA procedures compared with subsequent sessions (14.6% vs 38.9%, *P* < .001). The reason for BPA as a treatment modality did not differ between groups, including nonsurgical candidacy, post-PTE treatment, or patient preference.

**Table 2:** Procedural characteristics stratified by same-day versus different-day discharge.

Characteristic	All procedures (N = 262)	Same-d discharge		
		No (n = 173)	Yes (n = 89)	*P* value^a^
Initial BPA	74 (30.1)	61 (38.9)	13 (14.6)	<.001
BPA reason				.91
Not a surgical candidate	62 (23.7)	39 (22.5)	23 (25.8)	
Post-PTE	150 (57.3)	99 (57.2)	51 (57.3)	
Patient preference	50 (19.1)	35 (20.2)	15 (16.9)	
Location of intervention				
RUL	74 (28.2)	50 (28.9)	24 (27.0)	.91
RML	80 (30.5)	55 (31.8)	25 (28.1)	.55
RLL	117 (44.7)	81 (46.8)	36 (40.4)	.38
LUL	59 (22.5)	37 (21.4)	22 (24.7)	.31
LLL	83 (31.7)	51 (29.5)	32 (36.0)	.25
No. of segments with intervention	3 (2-4)	3 (2-4)	2 (2-3)	.08
Largest balloon used, mm	4 (3-5)	4 (3-5)	4 (4-6)	.053
Length of procedure, min	97 ± 23	101 ± 23	89 ± 19	<.001
Cardiac output, L/min	5.1 ± 1.2	5.1 ± 1.3	5.1 ± 1.1	.57
mPAP, mm Hg	32.7 ± 11.5	34.9 ± 11.0	28.5 ± 11.3	<.001
PVR, WU	5 (3-6)	5 (3-7)	4 (3-6)	.12
PVR, WU				.06
<4	69 (26.3)	37 (21.4)	32 (36.0)	
4-6	110 (42.0)	81 (46.8)	29 (32.6)	
>6	83 (31.7)	55 (31.8)	28 (31.5)	
Contrast volume, mL	162 ± 53	167 ± 54	154 ± 51	.08
Amount of radiation, mGy	594 (389-947)	556 (352-930)	634 (468-985)	.23
Type of hemostasis				.001
Manual	198 (76.2)	133 (77.8)	65 (73.0)	
Device closure	33 (12.7)	12 (7.0)	21 (23.6)	
Mattress suture	29 (11.2)	26 (15.2)	3 (3.4)	
Length of stay for same-d discharge, h	5 (4-6)	—	5 (4-6)	—
Length of stay for different-d discharge, d	1 (1-2)	1 (1-2)	—	—
Travel distance after discharge, mi	30 (16-54)	34 (17-65)	26 (15-45)	.70
Return within 30 d	14 (5.3)	7 (4.0)	7 (7.9)	.43

Values are either n (%), median (IQR), or mean ± SD.

Length of stay for different-day discharge is reported per hospital admission rather than per procedure. Some patients underwent sequential BPA procedures during a single admission; therefore, the number of admissions (n = 139) is less than the total number of non–same-day discharge procedures (n = 173). Postdischarge travel distance was available for 239 of 262 procedures; fewer than 4 procedures were missing data for the number of treated segments, largest balloon diameter, procedure duration, contrast volume, radiation dose, and hemostasis technique.

LLL, left lower lobe; LUL, left upper lobe; mPAP, mean pulmonary artery pressure; PVR, pulmonary vascular resistance; RLL, right lower lobe; RML, right middle lobe; RUL, right upper lobe.

a *P* value was determined by univariable mixed-effect logistic models.

**Central Illustration:**
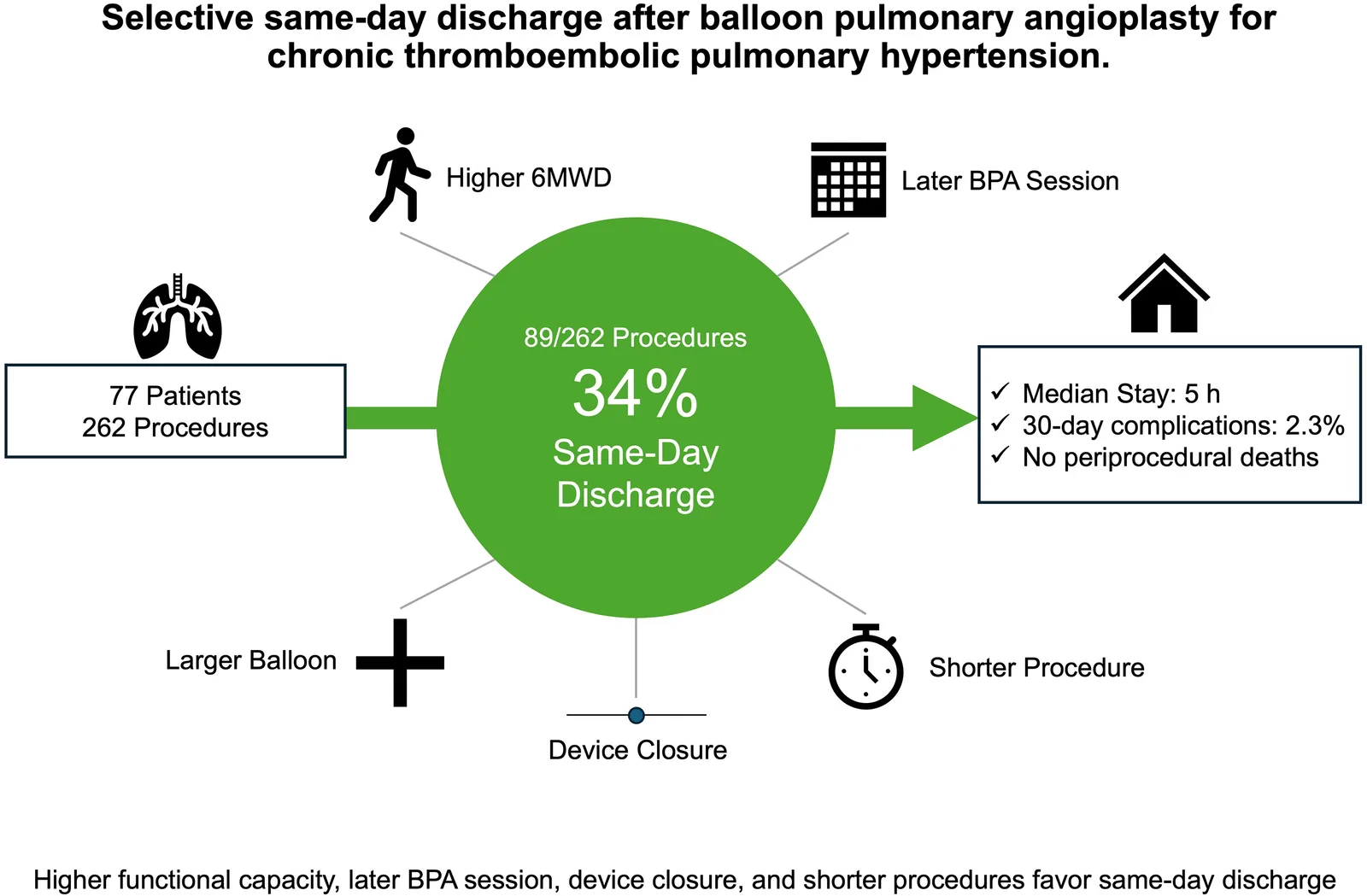
**Predictors and outcomes of same-day discharge after balloon pulmonary angioplasty for chronic thromboembolic pulmonary hypertension.** Among 262 BPA procedures performed in 77 patients, 89 (34%) resulted in same-day discharge. Higher baseline 6MWD, later procedural session number, shorter procedure duration, larger maximum balloon diameter, and use of a vascular closure device were associated with same-day discharge, which was achieved with a median hospital stay of 5 hours, a 30-day complication rate of 2.3%, and no periprocedural deaths. BPA, balloon pulmonary angioplasty; 6MWD, 6-minute walk distance.

The distribution of treated pulmonary segments (right upper, middle, and lower lobes; left upper and lower lobes) was similar, with no significant differences in the anatomic location of intervention. Though the differences were not significant, the number of segments treated per procedure was slightly lower in the SDD group (median, 2 [IQR 2-3] vs 3 [IQR 2-4]; *P* = .08) and the largest balloon size used was slightly larger in the SDD group (4 mm [IQR 4-6] vs 4 mm [IQR 3-5], *P* = .053).

Procedures resulting in SDD were significantly shorter in duration (89 ± 19 minutes vs 101 ± 23 minutes, *P* < .001) and associated with lower mPAP at the time of the procedure (28.5 ± 11.3 mm Hg vs 34.9 ± 11.0 mm Hg, *P* < .01). Cardiac output was similar between groups (5.1 ± 1.1 vs 5.1 ± 1.3 L/min, *P* = .57), and PVR did not differ significantly as a continuous variable (*P* = .12). When stratified by PVR category (<4, 4-6, >6 WU), PVR trended slightly lower in the SDD group (*P* = .06).

There were no significant differences in contrast volume, radiation dose, or postdischarge travel distance between groups. Hemostasis technique differed significantly, with device closure used more frequently in the SDD cohort (23.6% vs 7.0%), and mattress sutures less commonly used (3.4% vs 15.2%; *P* < .001).

Among procedures performed with SDD, the median length of stay was 5 hours (IQR 4-6). For those requiring hospitalization, the median length of stay was 1 day (IQR 1-2). Thirty-day return rates were similar between groups (7.9% vs 4.0%, *P* = .43).

Procedure-level data were largely complete. Postdischarge travel distance was available for 239 of 262 procedures (91.2%). Fewer than 4 procedures were missing data for each of the following: number of treated segments, largest balloon diameter, procedure duration, contrast volume, radiation dose, and hemostasis technique.

### Factors associated with SDD

In multivariable mixed-effects logistic regression analysis, several procedural and clinical factors were associated with SDD (Table 3) after adjusting for other covariates in the model and accounting for patient-level clustering. Higher preprocedure functional capacity, measured by 6MWD, was associated with greater odds of SDD (OR, 1.66 per 100 m; 95% CI, 1.06-2.60; *P* = .026). Initial BPA procedures were associated with markedly lower odds of SDD (OR, 0.13; 95% CI, 0.04-0.40; *P* < .001).

**Table 3:** Multivariable mixed-effects logistic regression identifying predictors of same-day discharge

Characteristic	OR (95% CI)	*P* value^a^
Age at surgery, y	1.01 (0.97-1.05)	.64
Required oxygen		.055
No	Reference	
Yes	0.32 (0.10-1.03)	
Preprocedure 6MWD, 100 m	1.66 (1.06-2.60)	.026
Initial BPA procedures		<.001
No	Reference	
Yes	0.13 (0.04-0.40)	
Hemostasis		.028
Manual	Reference	
Device closure	6.33 (1.59-25.23)	
Mattress suture	0.70 (0.12-4.05)	
mPAP, mm Hg	0.96 (0.91-1.02)	.16
PVR, WU		.056
<4	Reference	
4-6	0.38 (0.12-1.19)	
>6	1.64 (0.40-6.75)	
Largest balloon used, mm	1.44 (1.02-2.04)	.037
Length of procedure, 10 min	0.66 (0.53-0.83)	<.001

6MWD, 6-minute walk distance; BPA, balloon pulmonary angioplasty; mPAP, mean pulmonary artery pressure; OR, odds ratio; PVR, pulmonary vascular resistance.

a Multivariable logistic mixed model evaluated the association of same-day discharge with independent variables that were significant in the univariable analysis, as well as age and PVR group. Continuous variables are assessed per the unit specified in the table, centered at the following means: 61 years for age, 353 meters for 6MWD, 4.3 mm for balloon size, 96 minutes for procedural length, and 31 mm Hg for mPAP.

Device closure of the access-site was associated with SDD compared with manual compression (OR, 6.33; 95% CI, 1.59-25.23, *P* = .028). Larger balloon diameter was associated with increased odds of SDD (OR, 1.44 per mm; 95% CI, 1.02-2.04; *P* = .037), whereas longer procedure duration was associated with reduced odds (OR, 0.66 per 10 minutes; 95% CI, 0.53-0.83; *P* < .001).

Age, mPAP, oxygen requirement, and PVR category were not associated with SDD after adjustment, although a nonsignificant trend toward reduced likelihood of SDD was observed in patients requiring supplemental oxygen and those with intermediate PVR (4-6 WU).

### Complications

Despite differences in procedural characteristics and discharge patterns, overall BPA-related complication rates within 30 days of procedure were low (Table 4). Across 262 procedures among 77 different patients, BPA-related complications occurred in 6 cases (2.3%), corresponding to 6 different patients (7.8%). Complications included isolated events such as shortness of breath/anxiety, acute hypoxemic respiratory failure due to reperfusion injury, postoperative groin access-site bruising, pneumonia, and syncope with rectus sheath hematoma. Importantly, there were no periprocedural mortalities. At the procedure-level, complications occurred in 4 of 89 SDD procedures (4.5%) versus 2 of 173 non-SDD procedures (1.2%). Using a generalized estimating equation model, the difference was not statistically significant (OR, 4.2; 95% CI, 0.72-24.54; *P* = .11).

**Table 4:** BPA-related complications within 30 days

Complication	Per procedure (N = 262)	Per patient (N = 77)
Overall	6 (2.4)	6 (7.8)
SOB/anxiety	1 (0.4)	1 (1.3)
AHRF 2/2 reperfusion injury^a^	1 (0.4)	1 (1.3)
ED for bruising at the postoperative site R groin^a^	1 (0.4)	1 (1.3)
ED for wound check^a^	1 (0.4)	1 (1.3)
ED for multifocal pneumonia^a^	1 (0.4)	1 (1.3)
ED for syncope, rectal sheath hematoma	1 (0.4)	1 (1.3)

Values are n (%).

AHRF, acute hypoxemic respiratory failure; BPA, balloon pulmonary angioplasty; ED, emergency department; SOB, shortness of breath.

a Four of the 6 complications occurred in patients who were discharged on the same-day as their BPA procedure.

### Discharge patterns across sessions

**Figure 2:**
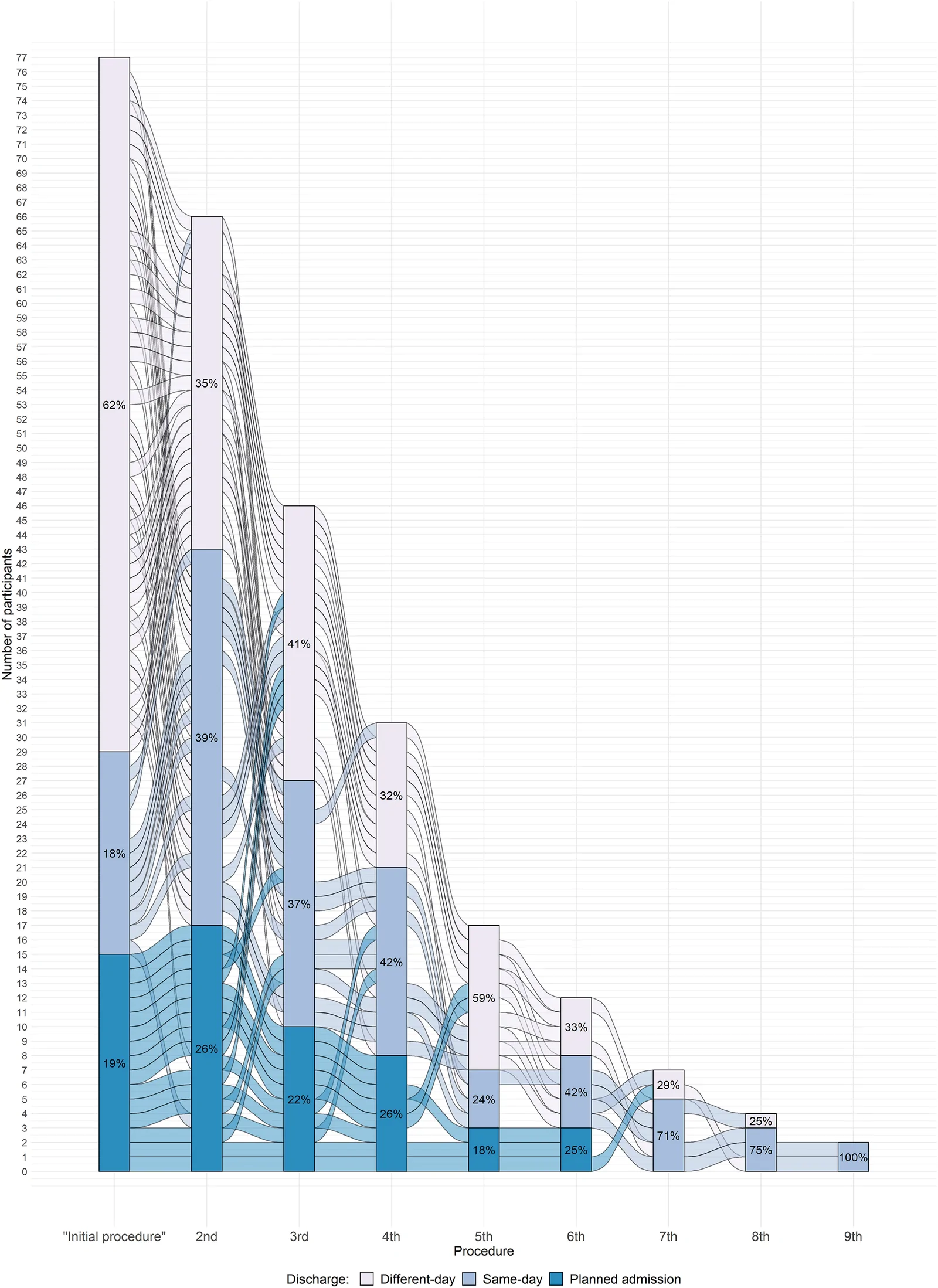
**Discharge status by procedure sequence.** Distribution of discharge disposition across sequential balloon pulmonary angioplasty sessions. Same-day discharge increased across later procedures, whereas planned admissions were concentrated during earlier sessions.

Discharge patterns also evolved across procedures for patients who received multiple BPA procedures (Supplemental Table S1). SDD occurred in only 18.2% of first procedures but increased across subsequent sessions, accounting for over 40% of discharges by the fourth and sixth procedures and more than 70% in later procedures (Figure 2). Planned admissions were largely confined to early BPA stages, while later sessions were increasingly associated with SDD.

## Discussion

In our cohort, approximately one-third of BPA procedures were followed by SDD in patients selected by a prespecified institutional eligibility algorithm. Among this group, short-term complication and readmission rates were low, with no periprocedural deaths. To our knowledge, this represents one of the largest contemporary US analyses evaluating SDD after BPA with a focus on patient- and procedure-level factors.

Factors that were associated with SDD included higher preprocedure 6MWD, subsequent BPA sessions in patients who underwent multiple procedures, the use of larger balloon sizes during BPA, and the use of device closure for hemostasis. On the other hand, initial BPA sessions and longer procedural duration were associated with overnight admission for observation.

Our institutional SDD practice has evolved with cumulative experience. Among the first 100 BPA procedures in this cohort, SDD occurred in 10%, compared with 49% (79/162) of the subsequent procedures. When restricted to procedures without planned overnight admission, the rate of SDD rose from 28% in the first 100 procedures to 57% (61/106) in subsequent procedures. This trajectory reflects both the changing composition of our case mix as the program matured and a growing operator and program familiarity with the postprocedural course, including a better understanding of which clinical events warrant prolonged observation.

The complication rates observed in our study align with those reported in contemporary US and international BPA cohorts,^6^^,^^17^^,^^18^ supporting SDD as an appropriate strategy when applied within a structured selection framework.

These findings suggest that both patient selection and procedural characteristics play important roles in determining the feasibility of SDD. Patients with higher preprocedure 6MWD were more likely to undergo SDD, suggesting that better baseline functional capacity and physiological reserve support earlier discharge after BPA.

A notable feature of our cohort is the substantial proportion of patients with prior PTE (51.9%), reflecting the role of our institution as a comprehensive CTEPH referral center performing both PTE and BPA. BPA in post-PTE patients is technically more complex than native-vessel intervention, as treatment is often performed in previously endarterectomized vessels where the endothelium has been denuded, potentially increasing the risk of vessel injury, hemoptysis, and reperfusion-related complications. The favorable outcomes observed in the post-PTE patients who underwent SDD, therefore, lend additional support to the feasibility of SDD when patients meet the institutional eligibility criteria, even in this inherently more complex subgroup.

Procedural factors also influenced discharge disposition. The use of device closure for hemostasis was associated with an increased likelihood of SDD, which may reflect more reliable hemostasis, reduced access-site bleeding, and earlier ambulation. Prior studies in interventional cardiology have demonstrated that vascular closure devices reduce time to hemostasis without an associated increase in vascular complications compared to manual compression, which may shorten the overall recovery time.^19^

Additionally, the use of a larger balloon size was associated with SDD. This finding seems counterintuitive, as a larger balloon size is not likely to directly reduce procedural risk during dilation. Instead, this finding may reflect the broader procedural context, as larger balloons may be preferentially used during later treatment sessions, or being used in more proximal disease, require less wire manipulation in the distal vasculature, avoiding the inherent risks associated. Importantly, however, the largest balloon used in this cohort was 6 mm, with intervention confined to the proximal segmental vessels. Generally, treatment of lobar vessels may be done surgically. When treatment of lobar or main vessels is being performed with BPA, larger balloon sizes are typically required and may carry an increased risk of reperfusion injury due to reperfusion of large territories. Therefore, despite the findings cited here, operators should be cautious that even larger balloon sizes in more proximal vessels may not have the same low adverse event rate.

In contrast, longer procedural duration was associated with overnight admission, potentially serving as a surrogate for procedural complexity or intraprocedural challenges that prompt a more cautious postprocedural management approach. In addition, the lower likelihood of SDD during initial BPA sessions may reflect higher starting PVR, relatively unknown anticipated procedure complexity, or clinical gestalt defaulting to the need for closer monitoring early in the treatment course.

In this cohort, the observed complication rate of 2.3% was comparable to rates reported in other contemporary single-center studies, which have described BPA-related complication rates of 2.6% to 4.7% and procedure-related mortality rates of 0% to 3.2%.^17^^,^^18^^,^^20^^,^^21^ Compared with earlier BPA experiences, which were characterized by higher rates of reperfusion lung injury and prolonged hospital stays, more recent studies have demonstrated improved safety profiles.

In the US, SDD following BPA, defined as discharge on the calendar day of the procedure without planned overnight observation, has only been reported in 1 descriptive single-center series of 59 sessions done in 29 patients over 3 years.^20^ In it, the authors reported no delayed complications or readmissions. We similarly observed low rates of BPA-related complications and no excess short-term adverse events among patients discharged on the same-day, albeit in a more select group of patients, and observed no differences in the rate of readmission between SDD patients and admitted patients. In addition, comparison of SDD patients and admitted patients in our study allowed identification of specific patient- and procedure-level factors associated with successful SDD, including functional capacity, procedural stage, hemostasis strategy, and procedural duration.

The increasing prevalence of BPA programs worldwide underscores the importance of understanding postprocedural safety and discharge practices as procedural volume grows. National and international data sets show steady growth in BPA volume over the past decade, driven by expanding indications and growing recognition of BPA as a central therapy for inoperable or residual CTEPH.^4^^,^^6^^,^^7^ As procedural demand rises, centers must consider the implications of postprocedural care pathways on safety, resource utilization, and patient experience.^11^^,^^22^ Our findings highlight patient- and procedure-level factors associated with SDD and may inform postprocedural protocols at similar US centers.

### Limitations

Several limitations warrant consideration when interpreting these findings. First, this study was conducted retrospectively at a single comprehensive CTEPH referral center with a modest sample size, and institutional practice patterns may differ from those at other centers. Second, discharge disposition was determined according to a prespecified institutional eligibility algorithm rather than through randomization, and patients undergoing SDD, therefore, represented a clinically and procedurally lower-risk subset by design. The favorable outcomes observed in this group should be interpreted as reflecting the performance of this selection framework in real-world practice rather than the safety of SDD across the broader BPA population. Because the eligibility criteria are explicit and reproducible, they may serve as a basis for protocol development at other centers with adaptation to local patient populations. Third, the small number of observed complications limited the precision of statistical comparisons between SDD and non-SDD groups, and the absence of a statistically significant difference should be interpreted as a function of low power rather than as evidence of equivalent safety. Fourth, complete data were unavailable for select baseline and procedural variables, as detailed in the Results section. Finally, evaluation of postprocedural events was confined to the early postprocedural period, and delayed complications or readmissions related to discharge disposition may not have been fully captured.

## Conclusion

Same-day discharge following BPA at an experienced comprehensive CTEPH referral center was feasible and not associated with an increased risk of short-term complications when guided by a prespecified eligibility algorithm. Clinical and procedural characteristics, including functional capacity and procedural stage, influenced discharge disposition. As BPA volume continues to increase, evidence-based SDD strategies may represent an important opportunity to optimize resource utilization. Prospective multicenter studies are needed to define standardized criteria for safe implementation.

## CRediT authorship contribution statement

**Kevin Jin:** Writing – review & editing, Writing – original draft, Data curation, Conceptualization. **Kelley Chen:** Writing – review & editing, Writing – original draft, Conceptualization. **Benjamin L. Magod:** Writing – review & editing, Writing – original draft, Conceptualization. **Zhiying Meng:** Writing – review & editing, Writing – original draft, Visualization, Validation, Methodology, Investigation, Formal analysis. **Heather Byrd:** Writing – review & editing, Visualization, Formal analysis, Data curation. **Abigail S. Baldridge:** Writing – review & editing, Validation, Supervision, Methodology, Formal analysis, Data curation, Conceptualization. **Daniel J. Romary:** Writing – review & editing, Data curation. **Benjamin H. Freed:** Writing – review & editing, Conceptualization. **Yasmin Raza:** Writing – review & editing, Conceptualization. **Maanasi Samant:** Writing – review & editing, Conceptualization. **Ruben Mylvaganam:** Writing – review & editing, Writing – original draft, Supervision, Project administration, Conceptualization. **Michael J. Cuttica:** Writing – review & editing, Supervision, Project administration, Conceptualization. **S. Chris Malaisrie:** Writing – review & editing, Supervision. **Stephen F. Chiu:** Writing – review & editing, Writing – original draft, Supervision, Project administration, Methodology, Conceptualization. **Daniel R. Schimmel:** Writing – review & editing, Writing – original draft, Supervision, Project administration, Methodology, Data curation, Conceptualization.

## Declaration of competing interest

Daniel R. Schimmel reports educational grant support from Medtronic, Edwards, and Abbott; educational grant support and consulting fees, and is on the advisory board of Boston Scientific; research grant support, consulting, and speaker fees from Penumbra and Stryker; research grant support and consulting fees from Inquis Medical; research grant support from Johnson & Johnson, Gradient, HeartFlow, and Pulnovo. Benjamin H. Freed reports research grant support from the Cardiovascular Medical Research & Education Fund. Michael J. Cuttica reports research grant support and speaker fees, and is on the advisory board of Bayer Pharmaceuticals, Johnson & Johnson, and United Therapeutics. S. Chris Malaisrie reports research grant support, consulting, and speaker fees from Edwards and Terumo; research grant support and consulting fees from Medtronic; speaker fees from AtriCure; and consulting and speaker fees from Artivion. All other authors reported no financial interests.

## Footnotes

To access the supplementary material accompanying this article, visit the online version of the *Journal of the Society for Cardiovascular Angiography & Interventions* at 10.1016/j.jscai.2026.105505.
