# Supplementary material for: Safety and Feasibility of Same-Day Discharge After Balloon Pulmonary Angioplasty for Chronic Thromboembolic Pulmonary Hypertension: A Single-Center Retrospective Study
Source: J Soc Cardiovasc Angiogr Interv. 2026 Jul 16;5(8):105505. doi: 10.1016/j.jscai.2026.105505 (PMC13491343; doi:10.1016/j.jscai.2026.105505)
Supplement: Supplementary file 1 [file mmc1.docx]

**Supplemental Table S1. Discharge disposition by procedural sequence.**

| **Discharge status for multiple procedures, no. (%)** | **1^st^** | **2^nd^** | **3^rd^** | **4^th^** | **5^th^** | **6^th^** | **7^th^** | **8^th^** | **9^th^** |
| --- | --- | --- | --- | --- | --- | --- | --- | --- | --- |
| Different-day discharge | 48 (62.3) | 23 (34.8) | 19 (41.3) | 10 (32.3) | 10 (58.8) | 4 (33.3) | 2 (28.6) | 1 (25.0) | 0 (0) |
| Same-day discharge | 14 (18.2) | 26 (39.4) | 17 (37.0) | 13 (41.9) | 4 (23.5) | 5 (41.7) | 5 (71.4) | 3 (75.0) | 2 (100) |
| Planned admission | 15 (19.5) | 17 (25.8) | 10 (21.7) | 8 (25.8) | 3 (17.6) | 3 (25.0) | 0 (0) | 0 (0) | 0 (0) |
